# Surgical training salvage during COVID-19: a hospital quality perspective

**DOI:** 10.1093/bjsopen/zrac019

**Published:** 2022-04-05

**Authors:** Oliver Luton, Katie Mellor, Catherine Eley, Osian James, David Brian Thomas Robinson, Luke Hopkins, Wyn Griffith Lewis, Richard John Egan, Welsh Surgical Research Initiative (WSRI) Collaborative, Andrew Beamish, Arfon Powell, Chris Brown, Simon Wood, Tarig Abdelrahman

**Affiliations:** 1 Health Education and Improvement Wales’ School of Surgery, Tŷ Dysgu, Cefn Coed, Nantgarw, UK; 2 Department of Surgery, University Hospital of Wales, Cardiff, UK; 3 Department of Surgery, Morriston Hospital, Swansea Bay University Health Board, Swansea, UK; 4 Post-graduate School of Medical Education, Swansea University, Swansea, UK

Dear Editor

Vicissitudes, including redeployment, elective cancellations, and remote educational events, have restricted training opportunities during the COVID-19 pandemic. Indeed, never before in modern times has surgical training been so vulnerable to such an existential threat. The first UK national lockdown started on 16 March 2020, largely halting elective surgery, including planned cancer treatment^1,2^. As hospitals came to terms with new rules of engagement, safe surgical pathways were developed together with a strengthened will to protect training^3^. To date, other than operative logbook caseload, evidence regarding the pandemic’s impact on training metrics is sparse^4,5^. Moreover, there have been no reports regarding individual hospitals’ ability to adapt to the new training environment. This study aimed to assess and compare training metrics, in particular those required for Certification of Completion of Training related to individual hospital units before and after COVID-19, to develop and explore a novel Unit Adaptability Score (UAS).

Fifty consecutive, nationally appointed Higher Surgical Trainees in General Surgery (GS) (median age 36 (range 29 to 46) years; 15 women and 35 men) were identified from one Statutory Education Body, Health Education and Improvement Wales. Primary effect measures comprised operative logbook cases, index procedures, and work-based assessments (WBAs). Outcomes were compared across two 12-month periods (period 1 (P1) 1 March 2019 to 29 February 2020 (non-COVID) *versus* period 2 (P2) 1 March 2020 to 28 February 2021 (COVID)) related to both trainees and training hospitals. Ranking hospitals into quartiles according to their median performance created a composite performance score related to the following metrics: total operative logbook numbers; procedures performed primarily by the trainee (surgical trainer scrubbed/surgical trainer unscrubbed/performed); index operations (appendicectomy, cholecystectomy, colectomy, inguinal hernia repair, laparotomy); and operative WBAs. An adaptability score was then created for each parameter, representing the change from performance baseline for each individual hospital during P2. Tertiary hospitals (THs) and district general hospitals (DGHs) were compared using a quartile-based approach, creating a scale of one to four, represented on a radar diagram.

Fifty GS HSTs undertook 191 six-month placements in 11 hospitals (TH = 2, DGH = 9). At an individual level during COVID-19, operative experience per placement fell 26.1 per cent (median 211 *versus* 156; *P* < 0.010) with a 32.1 per cent reduction observed in trainee primary surgeon experience (162 *versus* 110; *P* < 0.010). Regarding index procedures, cholecystectomy declined 45.5 per cent (11 *versus* 6; *P* = 0.027) and inguinal hernia 62.5 per cent (8 *versus* 3; *P* < 0.010). WBAs were similar (17 *versus* 13; *P* = 0.364). Despite relative equivalence during P1, median total operative procedures performed in DGHs (*n* = 65) were 40.9 per cent fewer than THs (*n* = 110, *P* < 0.010). The number of supervised trainer scrubbed/unscrubbed/performed cases was 25.4 per cent higher for trainees in THs during P2 (67 *versus* 50; *P* = 0.020). A radar plot (*Fig. 1*) of composite metrics provides a visual representation of the comparable effect of COVID-19 on training and revealed a wide performance differential and adaptability score between DGH (score = 16, radar chart coverage 44.4 per cent) and TH performance (score = 24, radar chart coverage 66.6 per cent). For data see the supplementary material,*Tables 1*–*4* and *Figs 2*–*3*.

**Fig. 1:**
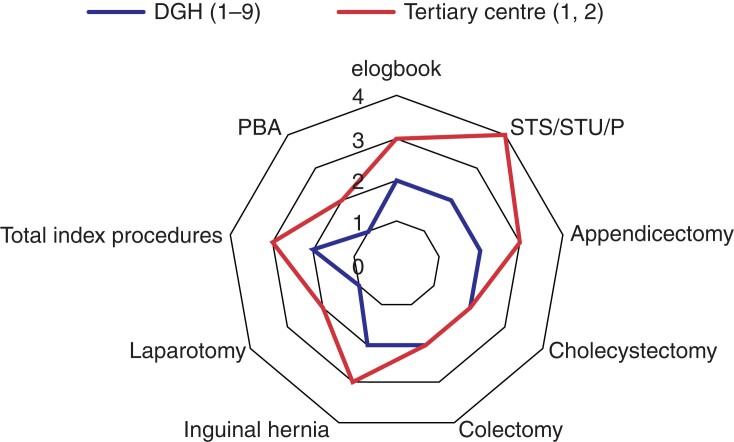
Radar plot representing the training gap between district general hospitals (DGHs) and tertiary hospitals (THs) due to the COVID-19 pandemic

TH performance score = 24, radar chart coverage 66.6 per cent. DGH performance score = 16, radar chart coverage 44.4 per cent. STS/STU/P, supervised trainer scrubbed/unscrubbed/performed; PBA, procedure-based assessment.

These findings provide the first objective data comparing surgical training metrics related to COVID-19 within General Surgery and the first to explore a new metric: the UAS—an adaptability index to benchmark training performance resilience in the face of existential threat. In broad terms, THs demonstrated the strongest adaptability and resilience to the threat of COVID-19, shielding education and maintaining trainee outputs within 5 per cent of normal historical performance. Quite why is unclear, and further research to examine hospital-level leadership, management structure, deprivation, and disease burden is desirable. Health board and hospital flexibility, faced with further pandemic-related existential threats and curriculum upgrade challenges, will be critical to health service recovery and training salvage.

## Supplementary Material

zrac019_Supplementary_Data

## Data Availability

Data, analytic methods, and study materials are made available as supplementary material.

## Collaborators

Andrew Beamish (Morriston Hospital, Swansea, Wales), Arfon Powell (University Hospital Wales, Cardiff, Wales), Chris Brown (Morriston Hospital, Swansea, Wales), Simon Wood (The Grange University Hospital, Cwmbran, Wales), Tarig Abdelrahman (University Hospital Wales, Cardiff, Wales).
