# Supplementary material for: Surgical training salvage during COVID-19: a hospital quality perspective
Source: BJS Open. 2022 Apr 5;6(2):zrac019. doi: 10.1093/bjsopen/zrac019 (PMC8982190; doi:10.1093/bjsopen/zrac019)
Supplement: zrac019_Supplementary_Data [file zrac019_supplementary_data.docx]

**Table S1.** Individual trainee outcomes from Non-COVID (P1) vs. COVID (P2) rotations.

|  | **Total P1** | **Total P2** | **P1 trainee median per annum** | **P2 trainee median per annum** | **p value** |
| --- | --- | --- | --- | --- | --- |
| **elogbook** | 6877 | 5292 | 211 (31-345) | 156 (22-284) | 0.003* |
| **STS/STU/P** | 4976 | 3969 | 162 (21-289) | 110 (12-243) | 0.008* |
| **Appendicectomy** | 711 | 626 | 24 (3-37) | 16 (0-46) | 0.057 |
| **Cholecystectomy** | 441 | 387 | 11 (0-43) | 6 (0-37) | 0.027* |
| **Colectomy** | 360 | 344 | 6 (0-42) | 6 (0-35) | 0.992 |
| **Inguinal hernia** | 304 | 156 | 8 (1-36) | 3 (0-16) | 0.003* |
| **Laparotomy** | 300 | 356 | 9 (1-22) | 9 (1-9) | 0.406 |
| **Total Index procedures** | 2116 | 1869 | 61 (15-122) | 52 (2-119) | 0.101 |
| **PBA** | 568 | 526 | 17 (0-38) | 13 (1-38) | 0.368 |

Results are reported as number of individuals or medians with ranges respectively. *indicates statistical significance. P1:1^st^ March 2019 - 29^th^ February 2020 (non-COVID). P2: 1^st^ March 2020 – 28^th^ February 2021 (COVID). STS/STU/P: Supervised trainer scrubbed/ Un-scrubbed/ Performed, PBA: Procedure based assessment.

**Table S2. District general hospital and Tertiary hospital trainee performance between P1 and P2 intervals.**

|  | **P1** | | | **P2** | | |
| --- | --- | --- | --- | --- | --- | --- |
|  | **DGH (1-9)** | **TH (1,2)** | **p value** | **DGH (1-9)** | **TH (1,2)** | **p value** |
| **elogbook** | 111 (29-223) | 102 (15-208) | 0.107 | 65 (20-172) | 110 (13-199) | 0.01* |
| **STS/STU/P** | 91 (1-163) | 63 (9-170) | 0.041* | 50 (12-155) | 67 (9-174) | 0.02* |
| **Appendicectomy** | 12 (2-24) | 12 (0-27) | 0.865 | 6 (1-23) | 11 (0-27) | 0.03* |
| **Cholecystectomy** | 6 (0-25) | 3 (0-21) | 0.126 | 2 (0-27) | 3 (0-32) | 0.94 |
| **Colectomy** | 5 (0-27) | 2 (0-25) | 0.435 | 3 (0-18) | 3 (0-25) | 0.17 |
| **Inguinal hernia** | 5 (0-24) | 2 (0-8) | 0.002* | 1 (0-11) | 2 (0-8) | 0.98 |
| **Laparotomy** | 4 (0-14) | 4 (0-17) | 0.818 | 4 (0-15) | 5 (0-21) | 0.97 |
| **Total Index procedures** | 37 (6-78) | 28 (5-63) | 0.177 | 22 (2-91) | 30 (5-66) | 0.13 |
| **PBA** | 8 (0-27) | 7 (0-15) | 0.061 | 6 (0-24) | 7 (1-22) | 0.24 |

Results are reported as six-month rotation medians with ranges. *indicates statistical significance. P1:1^st^ March 2019 - 29^th^ February 2020 (non-COVID). P2: 1^st^ March 2020 – 28^th^ February 2021 (COVID). DGH: District general hospital, TH: Tertiary hospital, STS/STU/P: Supervised trainer scrubbed/ Un-scrubbed/ Performed, PBA: Procedure based assessment.

**Table S3.** Compound performance scores of individual units (incorporating 9 metrics, with individual sores of 0-5)

| **P1** | | | **P2** | | |  |
| --- | --- | --- | --- | --- | --- | --- |
| **Hospital** | **Score** | **Radar chart coverage** | **Hospital** | **Score** | **Radar chart coverage** | **p-value** |
| DGH1 | 24 | 53.3% | DGH1 | 23 | 51.1% | 0.76 |
| DGH2** | N/A | N/A | DGH2 | 24 | 53.3% | N/A |
| DGH3 | 23 | 51.1% | DGH3 | 9 | 20% | <0.01* |
| DGH4 | 5 | 11.1% | DGH4 | 12 | 26.7% | 0.02* |
| DGH5 | 34 | 75.6% | DGH5 | 20 | 44.4% | 0.02* |
| DGH6 | 15 | 33.3% | DGH6 | 11 | 24.4% | 0.57 |
| DGH7 | 19 | 42.2% | DGH7 | 28 | 62.2% | 0.09 |
| DGH8 | 33 | 73.3% | DGH8 | 15 | 33.3% | <0.01* |
| DGH9 | 22 | 48.9% | DGH9 | 6 | 13.3% | <0.01* |
| TH1 | 17 | 37.8% | T1 | 21 | 46.7% | 0.16 |
| TH2 | 17 | 37.8% | T2 | 31 | 68.9% | <0.01* |

*Signifies statistical significance. DGH2** did not have available data for the P1 period due to a lack of trainee allocation during that time. DGH: District general hospital. TH: Tertiary Hospital.

**Table S4.** Adaptability scores of individual units

| **Hospital** | **Adaptability score** | **Radar chart coverage** |
| --- | --- | --- |
| **TH1** | 35 | 77.78% |
| **TH2** | 34 | 75.56% |
| **DGH7** | 30 | 66.67% |
| **DGH1** | 25 | 55.56% |
| **DGH6** | 24 | 53.33% |
| **DGH4** | 21 | 46.67% |
| **DGH5** | 11 | 24.44% |
| **DGH8** | 8 | 17.78% |
| **DGH9** | 6 | 13.33% |
| **DGH3** | 5 | 11.11% |
|  |  |  |

Scoring legend: 5 points- an increased figure P2 vs. P1, 4 points- no change (+/-5% from baseline), 3 points- 5-25% decrease, 2 points- 25-50% decrease, 1 point- 50-75% decrease, 0 points- >75% decrease. A total of 9 metrics were included in this score. DGH2 omitted from adaptability score table as no pre-COVID data allowing comparison. DGH: District general hospital. TH: Tertiary Hospital.

**Figure S1.** Radar plot showing the training gap between the highest (TH2) and lowest (DGH9) units during the COVID-19 pandemic.


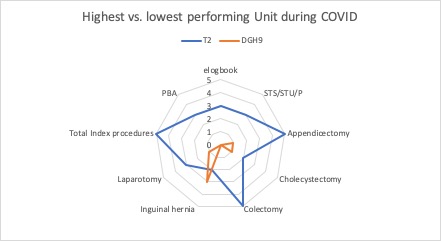


DGH: District general hospital. TH: Tertiary Hospital. STS/STU/P: Supervised trainer scrubbed/ Un-scrubbed/ Performed. PBA: Procedure based assessment.

**Figure S2.** Radar plot showing the difference between the most (T2) and least (DGH3) adaptable units during the COVID-19 pandemic.


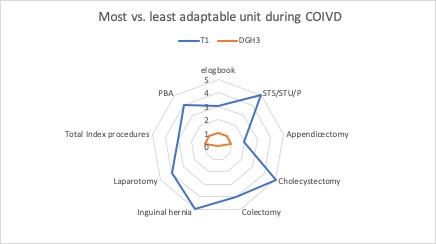


DGH: District general hospital. TH: Tertiary hospital. STS/STU/P: Supervised trainer scrubbed/ Un-scrubbed/ Performed. PBA: Procedure based assessment
